# Women’s Job Search Competence: A Question of Motivation, Behavior, or Gender

**DOI:** 10.3389/fpsyg.2018.00137

**Published:** 2018-02-13

**Authors:** Lucía I. Llinares-Insa, Pilar González-Navarro, Ana I. Córdoba-Iñesta, Juan J. Zacarés-González

**Affiliations:** ^1^Department of Social Psychology, University of Valencia, Valencia, Spain; ^2^Department of Developmental and Educational Psychology, University of Valencia, Valencia, Spain

**Keywords:** women’s employability, active job search, gender gap, work-life conciliation, unemployment of women

## Abstract

We examined motivation and behaviors in women’s active job search in Spain and the gender gap in this process. The current crisis in Spain and the increase in the number of unemployed people have revealed new inequalities that particularly affect women’s employability, especially the most vulnerable women. This paper addresses two exploratory studies: the first study analyzes gender differences in the active job search using a sample of 236 Spanish participants; the second study explores the heterogeneity and diversity of unemployed women in a sample of 235 Spanish women. To analyze the active job search, the respondents were invited to write open-ended responses to questions about their job search behaviors and complete some questionnaires about their motivation for their active job search. The content analysis and quantitative results showed no significant differences in motivational attributes, but there were significant gender differences in the job search behavior (e.g., geographical mobility). Moreover, the results showed heterogeneity in unemployed women by educational level and family responsibilities. The asynchronies observed in a neoliberal context reveal the reproduction of social roles, social-labor vulnerability, and a gender gap. Thus, women’s behavior is an interface between employment and family work, but not their motivations or aspirations. Our results can have positive implications for labor gender equality by identifying indicators of effectiveness in training programs for women’s job search, and it can contribute to designing intervention empowerment policies for women.

## Introduction

The financial crisis and neoliberalism in Spain have affected the labor market, especially for women. On the one hand, a functionalist social discourse legitimizes precarious jobs and blames women, resulting in an image of incompetence, laziness, and comfort (Kelvin and Jarrett, 1985; Noguera, 2000). On the other hand, women perceive themselves according to traditional gender stereotypes (Echebarria and González, 1999; Carter, 2014). This inequality, focused on training human capital (Becker, 1962), is based on the gender stereotype of ‘the good woman’ (Cornwall et al., 2008), who has to be efficient at work and in taking care of the family. This belief places women in a vulnerable social position, due to the lack of shared responsibility between men and women in Spanish homes (Del Río and Alonso, 2007). This happens to a greater extent to women in situations of exclusion who are inactive or unemployed and have great difficulties accessing employment. This situation is evident not only in the organizational (Alcañiz, 2015; Ingellis and Calvo, 2015) and gender (PNUD, 2014; ILO, 2015) scientific literature, but it is also a constant presence in social agents whose objective is to produce the transition to the ordinary labor market (e.g., accompanying technicians of social integration companies) (AERESS and FAEDEI, 2014). Based on this context and the studies initiated by Hakim’s (2006) preference theory, this paper analyzes gender differences in the active job search. Job search is a key competence in employability. More specifically, our purpose is to analyze whether women’s vulnerability in the access to employment is a motivational or behavioral issue.

Research on gender differences has shown the complex reality of the employment-unemployment relationship and its link to the sexual division of work and the work–family balance (Hoffman and Moon, 2000). Regarding employment, a large number of studies have analyzed the professional choices of women and men and what has been called occupational horizontal segregation. These studies show that women focus on professional activities directly related to the role and attributions of their gender (Yerkes, 2010). Another group analyzed the differential conditions of hiring, the workday, or the salary; this situation is known as occupational financial segregation (Palacio and Simón, 2006). A third group analyzed the occupational vertical segregation, which focuses on the difference in status between men and women in the workplace (PNUD, 2014).

These studies indicate that there are still stereotyped professional (and academic) choices and invisible mechanisms of discrimination in the working world. These mechanisms hinder the development of women’s professional careers and place them in a situation of inequality with respect to men. They also reveal a desolate employment panorama, especially for women with precarious jobs or women who have not yet accessed a job or have failed to access one (Davia, 2014): battered women, women in a situation of social exclusion or at serious risk (e.g., ethnic minorities), or women who are unemployed and have major difficulties in joining the ordinary labor market (e.g., ex-drug addicts in the process of rehabilitation and social reintegration).

Therefore, this situation perpetuates unequal access to and control of the resource of ‘employment,’ leading to greater poverty for women. Considering the clear inequalities in the access to employment, and thanks to the social movement toward gender equality in the labor context (Commission of the European Communities, 2000), positive discrimination policies have been developed for women (Lombardo and León, 2014). Since 1992, the European Union has pointed to the need to implement strategies to facilitate the inclusion, protection, and promotion of women in the labor market in order to achieve the development and stability of countries’ economies. Furthermore, in connection with the objectives of social cohesion and sustainable development in Europe, the Lisbon Strategy presents increasing women’s activity and employment rates as key objectives (Rodríguez, 2011). Currently, the International Labor Organization, in addition to other entities, highlights the need to incorporate gender equality issues in the promotion of employment (ILO, 2016).

However, these policies have not produced important changes in women’s current situation in the workplace (e.g., European Commission, 2010; Instituto Nacional de Estadística [INE], 2017). Therefore, the Agenda 2030 for Sustainable Development, adopted by the United Nations in 2015, reaffirms the importance of the gender gap in incorporating women into employment (ILO, 2016). In this regard, gender and organizational literatures have focused mainly on highlighting gender differences; however, it is necessary to identify gender indicators of this conflict. In this paper, we intend to take a further step in order to find out where the gender gap lies, based on Bronfenbrenner’s bioecological model. This analysis will try to respond to a series of unresolved questions in the literature: Can the behaviors and/or motivations toward the job search produce gender differences? Does the gender gap support the neoliberal ideology? This information would allow us to contribute to identifying the mechanisms underlying the job search gender gap. From a practical perspective, understanding some job search indicators can be useful in designing intervention programs (e.g., labor insertion itineraries for women) and opening up a debate to differentiate potential sources of the gender gap in the access to employment.

### Theoretical Background

The current crisis and the persistence of vertical, horizontal, and financial segregation in the labor market have increased the number of unemployed people and revealed new inequalities (Torns and Recio, 2012) that particularly affect women’s unemployment. International Organizations, States, NGOs, and stakeholders develop intervention programs and actions to reduce the gender gap in the labor context. Despite these efforts, the results have not been satisfactory, and the gender gap is evident (Fridkin and Kenney, 2007), especially in unemployed women actively seeking employment and / or with precarious and unstable jobs (Nadasen, 2012). This situation indicates that there has been insufficient analysis of the actual dimensions of women’s access, preservation, and professional development in the workplace.

This study relies on an integrative concept of employability (Llinares et al., 2016), based on Bronfenbrenner’s bioecological model (Bronfenbrenner, 2005; Bronfenbrenner and Morris, 2006), and placing an emphasis on its four components: proximal processes, biopsychological characteristics of a developing person, the parameters of the ecological context, and the temporal dimension. This framework is characterized by considering employability as a meta-competence, but also as a socially constructed historical category subject to socio-historical and institutional factors. Currently, the debate on employability involves the definition of its components (Rothwell et al., 2008), or the set of indicators (Llinares et al., 2016) that make it possible to identify the competences to be developed, the learning contents to be transmitted, and the elements to be evaluated (for example, Buchmann, 2002; Fugate et al., 2004; McQuaid and Lindsay, 2005). One of the most widely used classifications is the one described by Fugate et al. (2004), which considers three key dimensions of employability: professional identity, personal adaptability, and human capital. Professional identity refers to the work context and how to manage employment opportunities based on variables such as motivation. Motivation, according to McArdle et al. (2007), drives the job search activity. This motivation to active job search is interpreted from Hogan et al. (2013)’ model of employability as a willingness to hard work by employers, and then as an antecedent of employability. Personal adaptability refers to the willingness to learn, and human capital is focused on personal factors (e.g., age). This perspective links motivational aspects to the job search (McQuaid and Lindsay, 2005), as one of the four pillars of the European Employment Strategy (McQuaid et al., 2005). In this study, we will use the personal motivational variables relevant to job search processes. Thus, we will focus on variables that are clear precursors of the job search, the intention to search for a job, and job search behaviors, as well as gender differences in these variables, taking into account the gender differences and the heterogeneity of the group of women. The purpose is to investigate whether or not men and women have similar motivations and use similar behaviors to solve the unemployment situation.

Because women are one of the most vulnerable groups in accessing employment, individual differences and experiential trajectories are keys to distinguishing between the employed and the unemployed (Formichella and London, 2005; Weller, 2009). In addition, they can be combined with the concept of family in Spanish culture, characterized by an egalitarian family model in explicit dialogs, but based on the conviction that women should prioritize the family in everyday life (Fernández and Tobío, 2006). Thus, these two contradictory gender roles (Martínez et al., 2011) warrant more investigation about the gender gap (Lips, 2013), which as Allen et al. (2015) suggest, involves work-family conflict.

Currently, there has been considerable progress in the theoretical and empirical analysis of employment and family compatibility from different social science areas (Muffels et al., 2002). In 1983, Friedan analyzed the identity problems that arose when women with family responsibilities initiated a job and had to perform both tasks. Along these lines, some studies (e.g., Hakim, 2002; Grimshaw and Rubery, 2015) analyzed whether all women have a genuine choice between family and a job. Hakim (2005) analyzed family life styles and established three styles depending on the type of job conciliation preferences (Hakim’s preference theory): (a) Adaptive women: women who attempt some combination of employment and family without prioritizing either of them in an egalitarian family where both partners work and there is equity between the family roles. Adaptive women are the largest group among women who prefer occupations that facilitate a more even work–family balance than part-time work and temporary work; (b) Women whose lives are focused on their careers. Work-centered women are a minority; they focus on competitive activities, remain childless, and prioritize their jobs like men do; (c) Women whose attention is focused on the family and home. Family-centered women are also a minority, and they avoid paid work after marriage unless the family is experiencing financial problems. The Hakim (2006) results show that preference typologies were not evident in the past, but in today’s society they are present and show women’s heterogeneity. These preferences are linked to motivation (Leahy and Doughney, 2006), and as Koen et al. (2016) suggest, motivation is crucial in the use of exploratory job search strategies. Hakim (2005) pointed out that, unlike other nationalities, Spanish women show a preference for the third option because they are motivated to devote themselves to the family and household; thus, most women with children choose behaviors included in the third option. These data show the difficulty of balancing work and family (because there is a lack of regulation of the family-work balance), the gender effects on women’s access to the job market, and women’s social care role in the Spanish context. In this line of research, based on the aforementioned Hakim (2005) perspective and trying to understand divergent patterns of male–female employment, in this study we consider the family-job balance of men-women and whether the job conciliation preferences are the same for men and women. Thus, we intend to take a step forward in analyzing women’s employability and studying whether there are gender differences in motivation and job search behavior.

### Motivation and Active Job Search Behaviors

Motivational and behavioral approaches to the analysis of the job search process are two of the most widely used approaches by OECD employment services (Organization for Economic Co-operation and Development) (Escot and Fernández, 2013), and motivation enhancement is included in the effectiveness of job search intervention programs (Liu et al., 2014).

Motivation is the psychological process that promotes the behavior’s purpose and direction (Kreitner, 1995) and plays an important role in the job search process (Van Dam and Menting, 2012; Zimmerman et al., 2012). Moreover, Koen et al. (2016) pointed out, based on self-determination theory (SDT; Deci and Ryan, 1985, 2000; Gagné and Deci, 2005), that different types of job search motivations (from autonomous to controlled) impact job search behavior. Individual autonomous motivation is related to an intrinsic goal (Sheldon et al., 2003), whereas controlled motivation toward the goal has to do with the external environment (Sheldon and Elliot, 1998; Moran et al., 2012). Controlled motivation can be useful to explain the importance of the social context in job search behavior because complying with others’ demands involves external regulation (Vansteenkiste et al., 2004).

Motivational theories focus on explaining job-seeking behavior by using determinants of motivation. These theories have their origins in different fields of psychology. In a recent meta-analysis, Liu et al. (2014) pointed out that the opportunity to be employed was greater when the unemployed person participated in some type of training. Moreover, training was more effective when motivation techniques were used. In this context, some research has focused on the role motivation plays during the job search and in designing programs to improve job search success (Da Motta and Gabriel, 2016). Some of the elements outlined in cognitively oriented programs are (Aramburu, 2003): perceived control (Piqueras et al., 2016), attitudes (Kulik, 2001), the development of expectations of professional integration (eg., Feather, 1992), locus of control (Caliendo et al., 2015), and self-efficacy (McGee, 2015). These elements are described by the Theory of Planned Behavior (Ajzen, 1991), which has also been applied to job seeking (Fort et al., 2015).

**Perceived control** implies a belief in one’s ability to influence and develop the events that occur in life (Skinner, 1996). Increasing control can stimulate people to exercise control over their life events. Similarly, in a job search context, a greater perception of control over employment generates new job search intentions (Offerhaus, 2012; Caliendo et al., 2015) and stimulates the search for resources to avoid unemployment (Infurna et al., 2016). However, in the opposite direction, the loss of perceived control due to unemployment can cause damage to the human (skills) and social capital needed in the search for employment (Fugate et al., 2004). It can also generate feelings of helplessness and negative emotional states (for example, anxiety, stress, depression, for reviews, see Seligman, 1975), with negative consequences for work behavior (Lent et al., 1994, 2002; Wanberg et al., 2005) and a decrease in the active job search.

**Expectations of socio-professional integration** have usually been addressed as expectations of success (for a review of these models, see Feather, 1992). It refers to the subjective estimation of a person’s global possibilities of getting a job (Locke and Latham, 2002; Saks, 2006). The expectation of success is a good predictor of employment (Montilla, 2002; Piqueras et al., 2016) and, thus, of an active job search.

**Locus of control** is a perception about the influence of an event on behavior or on one’s relatively permanent personal characteristics (Rotter, 1966; Soriano and Lozano, 2006). This construct has also been widely studied in the context of unemployment (Caliendo et al., 2015; Cobb-Clark, 2015), and it is related to beliefs that getting a job will depend on one’s behavior. Hence, an unemployed person with an external locus of control will think that finding a job will depend on influential or powerful people or on luck or destiny (Rotter, 1966), whereas job seekers with an internal locus will consider that more effort leads to a higher probability of finding a job.

**Self-efficacy** is a person’s confidence in his/her ability to carry out a specific behavior (Bandura, 1986). In relation to a job search, the results obtained are inconsistent (Liu et al., 2014). However, Liu et al. (2014), in a meta-analytic study, showed that job search interventions are more effective when job search self-efficacy is developed, among other attributes.

**Attitudes** are the person’s evaluation of objects, events, and other people. Attitudes have been studied in job seeking (Fort et al., 2015) and, specifically, unemployment attitudes in job search (e.g., Kulik, 2000). However, the paths between the job search attitude and job search behavior are not congruent. To explain these discrepancies, some external and moderating variables have been proposed, such as the ethnic group (Van Hooft et al., 2004) and family situation (Van Hooft et al., 2005).

On the other hand, some research suggests that predictors and consequences of employment depend on the specific job search behaviors (Saks, 2006; Van Hoye et al., 2009). Job search behaviors are a self-regulatory and dynamic process that begins with the identification of an employment goal that activates job search behavior (Kanfer et al., 2001). In this process, Blau (1994) distinguished between preparatory and active job search behaviors. The sequential process begins with the preparatory phase; in this phase, individuals gather information about a potential job through, for example, job sites. Subsequently, job seekers begin active job search behavior (i.e., contacting employers).

Given that preparatory and active job search behavior is a stronger predictor of employment outcomes (Saks, 2006), the behavioral approach has mainly been developed in intervention programs. These programs are designed to increase and diversify job search behaviors (Stidham and Remley, 1992; Aramburu, 2003). Thus, we want to find out what actions are undertaken by unemployed people to find a job. Furthermore, previous literature reveals that behaviors differ according to gender roles (Van Hooft et al., 2006). For example, Eriksson and Lagerström (2012) showed gender differences in job searchers’ choice of search area and found that women were more restrictive than men in their choice of search area. In this line, Kunze and Troske (2015) studied gender differences in job search behavior over life-cycle and they found that job search behavior were different between men and women during childbearing years. These differences can be explained by gender role theory. This theory shows that gender differences in behavior are related to gender stereotypes, which lead to control over behavior and can influence decisions even without visible external pressure (Barbulescu and Bidwell, 2013). Even though some research points out these gender differences in job search behaviors, there is little direct evidence about the gender differences in job search behavior (Eriksson and Lagerström, 2012). This lack of interest of unemployed women (Kulik, 2000, 2001) has been explained by the prevailing assumption that the centrality of work is lower for women than for men (Kaufman and Fetters, 1980). According to the Role Theory Approach, a person’s behavior is strongly influenced by social norms and expectations of the role partners (Eagly, 1987). In this regard, through a socialization process (Kulik, 2001), the role of maintaining full responsibility for the children and household (Crosby, 1987) is emphasized in women, and the employee role is secondary (Eccles, 1987). Bianchi et al. (2000) reported that married women spend almost twice as much time on housework as married men do.

To study the motivational and behavioral aspects of the job search by gender, it is important to understand the gender gap in labor. However, there is a scarcity of studies investigating gender-based motivational and behavioral differences in the job search. It is important to fill this gap for several reasons. First, as mentioned above, previous research on the job search by gender has been carried out on these issues separately. Generally, these results and conclusions draw on an atomistic understanding. In order to avoid this, we need studies that examine the motivational and behavioral aspects of the job search together. Therefore, using Hakim’s preference theory, we argue that gender differences exist in the job search behavior, but not in the motivation for the job search. We believe that these behaviors are different in men and women because social images and stereotypes of traditional gender roles (López-Ibor et al., 2010) blame women for ‘family abandonment’ (Alberdi, 1999) or ‘defamilization’ (Rodríguez, 2011), and they view the woman’s job as a secondary economic source (Gómez, 2008).

Second, there is no unanimity in the results obtained. Some studies demonstrate that there are gender differences in motivational and behavioral aspects, and other do not. Some studies have showed that gender was not related to job search motivation (Vansteenkiste et al., 2005). Moreover, while previous studies (e.g., Kulik, 2000) showed evidence of gender-based differences in unemployment attitudes, in a later study Kulik (2001) proposed that there is a decrease in gender differences in unemployment attitudes currently in light of women’s changing career orientations. Other studies show that women are not more sensitive to social pressure than men and there are not important differences in job search behavior (e.g., Cross and Madson, 1997; Faberman et al., 2017). Kanfer et al.’s (2001) meta-analysis showed that gender has only a small influence on job search behavior; they show that men were more likely to engage in job seeking than women. In other hand, some studies have showed differences in the job search strategies (e.g., formal and informal strategies), job search intensity, job mobility, job preferences, … (Nicholson and West, 1988; Keith and McWilliams, 1999). Kulik (2001) suggested that educational level could explain these gender differences; thus, the higher educational level the less gender differences. Currently, the increase of educational level in Spain has changed the women’s job search. This educational development has changed the women’s career orientation and has brought them closer to man; in this sense, Kostal and Wiernik (2017), in a meta-analytic investigation about career orientations, showed slight gender differences in current career orientation. The rapprochement between men and women in educational level and their career development leads similarity in interest and motivations toward the job search. However, the women and men behavior are affected differently by households as it is explained above. Due to the social pressure, women are more likely than men to balance job search behavior with care responsibilities (Van Hooft et al., 2005). These perceptions of social pressure should be stronger predictors of behavior (Ajzen, 1991). Then, gender role affects job search behavior influenced by the perception of different gender social responsibilities (Martínez, 2006). Therefore, we hypothesize that Spanish women have the same work motivations as men in the active job search (hypothesis 1), but that there are gender differences in the job-seeking behavior. Women try to combine their work in the workplace and their work at home (hypothesis 2). In order to improve our understanding of gender differences in the job search hypothesized, it is necessary to study heterogeneity in the trajectories of unemployed women.

### Heterogeneity in the Trajectories of Unemployed Women

In the present study, and following Hakim (2001), in order to understand women’s job search, we assume that women are heterogeneous. Gender studies on employment/unemployment use sex as a variable to segment the sample (Suárez, 2004). They start from its conceptualization as a symbolic construct that contains attributes assigned to people based on gender. Thus, they analyze and explain the experiences of men and women from a relational perspective. Gender is used to legitimize inequalities in a neoliberal labor context where the variety of their determinations becomes invisible. These mechanisms linking work and family life have been identified, first, as a segregationist view and, second, as a concept of work-life boundaries (Santos, 2015), determinants that are combined in the formation of women’s identity and subjectivity. Therefore, using the elements of occupational segregation, and based on this new perspective on the nature of this continued diversity, the second objective of this paper is to examine the heterogeneity of the group of women in the motivational and behavioral aspects of their active job search. Several attributes may help to explain the women’s heterogeneity. Yerkes (2010) and Ayllón and Gábos (2017), among others, found effects of individual differences in educational level, marital status, and motherhood on women’s employment patterns. In relation to the educational level, there is a general conviction that the job search differs according to the level of people’s education. The increase in the educational level is related to changes in the traditional work role, giving greater value to work (Legazpe, 2015). In this regard, some research has shown that the educational level is related to women’s job decisions (Legazpe, 2015) and work behavior (Abdel-Mowla, 2011).

On the other hand, the relationship between women, work, and marriage is a controversial key issue. In the present study, child rearing and marital status are defined as family responsibility. The literature shows that childbearing and marital status have important consequences for women’s employment (Seager, 2000) and affect the job search (Van Hooft et al., 2005); for example, women usually leave their jobs (Hersch and Stratton, 1997) or restrict their job search to nearby areas under their control, even though they receive fewer job offers and lower employment and/or wages (Eriksson and Lagerström, 2012). Thus, women’s professional and work opportunities decrease (Gatrell, 2007, 2008; Haynes, 2008), which makes it possible to state that the gender gap in domestic work remains unchanged (Santos, 2015). Moreover, other authors have proposed **age** (Torns and Recio, 2012; Ming and Lin, 2013), **perceived social benefits** (Anker, 1997), and **nationality** (Adib and Guerrier, 2003; Motellón and López-Bazo, 2014) as being responsible for the differences in female employment. In this direction, Davia and Legazpe (2014) found that younger Spanish women have non-traditional work-home careers, whereas immigrant women in Spain have jobs that do not necessarily match their skills (Alonso-Villar and del Río, 2010). Therefore, this occupational segregation could affect the controlled motivation described by Deci and Ryan (2012), women’s attitudes and control in their job search, and their job search behaviors. In addition, based on gender role theory, employed women have a secondary economic role, and based on Hakim’s theory, they could have behavioral differences in the work–family conflict. Thus, women look for a job when there are economic problems in their family. Finally, when women weigh the social and economic benefits, they prefer to give priority to family life.

Taking these arguments into account and because research focused on heterogeneity women job search is very recent, we propose two exploratory hypothesis. We hypothesize that **family responsibilities and educational level** will show differences in the motivation for an active job search (hypothesis 3); however, there will be no differences in the active job search behavior (hypothesis 4) when **controlling for age, nationality, and the social benefits received.**

## Materials and Methods

We decided to carry out two studies to explore this gender situation. Study 1 aims to analyze the job search process by gender, as it is the situation that produces gender differences and distinguishes the activities people perform (Reskin and Padavic, 1994). Study 2 goes one step further and analyzes the heterogeneity in women’s employment with regard to their motivational and job-seeking behavior patterns. We want to detect the influence of gender social norms on unemployed women’s patterns, based on educational level, family responsibilities, and the presence of children (Yerkes, 2010) as starting points, but controlling the influence of the women’s age, nationality, and whether they receive social benefits. To address these objectives, the design of the two studies was descriptive and cross-sectional, and we used semi-structured interviews and self-report questionnaires, which were considered the most suitable methods to collect the data.

### Study 1: Motivational and Behavioral Aspects of Active Job Search by Gender

#### Participants

The current research is a descriptive study with purposeful sampling. Specifically, it started with theoretical sampling in order to decide which data were going to be analyzed and what kind of sample was relevant for this purpose. The selection criteria for the sample were as follows: Unemployed people searching for a job, workers with precarious employment who want to change their jobs, in other words women or men engaged in job seeking. Furthermore, we collected information about their marital status and whether the individuals had children or not. Subsequently, we delimited the collectives under study, and we carried out an opinion sampling where we selected the informants following the criterion of participation in the Valencian Employment and Training Service of the Valencian Community Government of individuals searching for employment or better jobs. The sample consisted of 236 participants (116 women, 49.2%, and 120 men, 50.8%) from the region of the Valencian Community, Spain. In the women’s sample, the average age was 32.6 (*SD* = 12.04), and 36% of them had children. Participants had different educational levels: elementary school (26.8%) and university graduate (45.5%). Moreover, 47.2% received some type of social benefits, and the respondents were mainly Spanish (62.2%). In the men’ sample, the average age was 34.13 (*SD* = 11.66), and 39.2% had children. In terms of their level of education, the majority did not have a university degree (61.4%). Some of them (42.5%) received a minimal social benefit. Regarding their occupational characteristics, 19.3% had contracts. With regard to nationality, 61.2% were Spanish.

#### Procedure

The sample was a convenience sample collected over a period of approximately 2 months in 2013. The authors coordinated the data collection. The questions were asked by the researchers, and participants filled in the questionnaires, which were gathered on site. Participants were informed that all data would remain anonymous and be used only for research purposes; that there were no right or wrong answers, and that they should answer the questions as honestly as possible (Podsakoff et al., 2003). All the individuals participated voluntarily in the study. In addition, the research followed Resolution No. 008430 of October 4, 1993, which establishes the scientific, technical, and administrative rules for health research, and protects the integrity and privacy of the individuals in the research. Additionally, we followed Law 1090 of 2006, which regulates the profession of Psychology and dictates the Code of Ethics and Bioethics. Moreover, this study fulfills the criteria for methodological rigor proposed by Guba and Lincoln (2002) to ensure the validity and reliability of qualitative research.

#### Instruments

To analyze the motivational and behavioral factors of the active job search, we used an instrument with three parts. The first part includes the participants’ socio-demographic information (age, marital status, sex, educational level, number of children, social benefits, and employment status). The second section provides information about the motivational aspects of the active job search: attitudes toward the job search, believing in one’s own competence, expectations of success in the job search, expectations of control, and attribution of causality. The third part requests information about the person’s job-seeking behavior.

##### Unemployment attitude

To evaluate the attitude toward unemployment, we used an adaptation of the Sánchez (1979) semantic differential scale. The scale has 10 pairs of antonyms that people have to score on a Likert scale from 1 to 6 (e.g., “good-bad”). The heading of the questionnaire was “unemployed person.” Cronbach’s Alpha was 0.99.

##### Expectations of socio-professional integration

It assesses competency and expectations of success in the job search. We used the Spanish Cuestionario de expectativas de inserción socioprofesional (CSQ) (*Questionnaire of expectations of socio professional integration*) (Figuera, 1994). The questionnaire contains 20 items with a six-point Likert scale, and answers can range from 1 (never) to 6 (often). This scale is composed of four factors: outcome expectations (α = 0.63) (e.g., ‘hardly worth seeking work’ or ‘worry because it is hard to find’); job search self-efficacy (α = 0.62) (e.g., ‘I trust in my own abilities, and I know I’ll find a job’); achievement motivation (α = 0.62) (e.g., ‘I expect to spend a significant part of my time building a career and developing the necessary skills to advance in it’); and role conflict (α = 0.68) (e.g., ‘You lack the necessary skills to practice as a professional’).

##### Perceived control of employment

Information about the worker’s perception of control over employment success was evaluated using the Escala de percepción de control del empleo (*Scale of perceived control of employment*) (Villar, 1991). This scale contains three items (e.g., “I believe that if a person really searches for a job, he/she will find one”) assessed on a five-point Likert scale of influence (α = 0.6) that evaluates the perception of controllability.

##### Locus of control in employment success

To evaluate the locus of control in employment success, we used the ATRI Scale (Figuera, 1994). This scale evaluates the tendency toward internality (e.g., “I have no ability to express myself and communicate”)/externality (e.g., “to change the government’s economic policy”) in employment success. The individual has to evaluate different factors that can explain the success of a satisfactory professional status. Each scale has six items with a five-point Likert scale (from no influence to a high influence) (internal attribution: α = 0.57 for internal locus; α = 0.91 for external locus). Because the alpha Coefficient was < 0.6, we calculated Guttman’s split-half reliability coefficient and Spearman-Brown’s split-half reliability coefficient, and the parallel model was > 0.70.

##### Active job search behaviors

To evaluate the Job-seeking behavior, we used a test of free association of words (Rotter, 1996). The person had to spontaneously write 5–10 words or expressions about job search behaviors.

#### Data Analysis

Basic descriptive statistical analyses were carried out to obtain the means and standard deviations for the involved variables in the question about job search behaviors. Moreover, reliability analyses were conducted to show the internal consistency of the scales used (by obtaining the Cronbach’s α). Finally, *t*-test analysis, ANOVA, chi-square, contingency coefficient, Phi, Cramer’s V, and MANCOVA were carried out to study the differences.

To analyze the job search behaviors, we used a free association test. To analyze the content, we created a categorization using the following criteria: comprehensiveness, mutual exclusion, consistency, relevance, objectivity and loyalty, and productivity (Fox, 1981). In this case, based on the way it is explained in the qualitative methodology, and considering that the researcher is the one who analyzes the results of his/her research, one of the basic elements to take into account is the elaboration and distinction of topics in order to collect and organize the information into categories. For this purpose, we differentiated between categories related to a topic, and subcategories, which divide this topic into micro-aspects based on inductive coding techniques. In this case, we decided not to perform any coding prior to the data collection, in order obtain the codes directly from the data (Cisterna, 2005). Therefore, following Anguera (2003), the starting point was the development of a repertoire or list of behavioral traits (reality) carried out in a job search. In order to form groups with similar behaviors, we took into account the classification of the employability indicators of Llinares et al. (2011) and the review of job search behavior (Van Hoye, 2013). A group of experts gave a provisional name to these similarities, thus generating a provisional taxonomy in which each category was defined (Moreno et al., 2002). With this categorization, our purpose was to ensure internal homogeneity between the different items classified in each category and an external homogeneity between categories (Anguera, 1986). Next, we took the list of behaviors again and assigned each behavior to the provisional groups already made. Subsequently, we analyzed whether there was an adequate degree of homogeneity between the registered behaviors without making modifications. Therefore, we assured a comprehensive and mutually exclusive system in each category.

Two independent judges codified the items in the selected categories. The consistency and reliability of the classification of job search strategies were obtained through agreement between the two judges (Krippendorff, 1990, 2013). The Cohen’s Kappa value exceeded 0.81, indicating a high level of agreement (Altman, 1991). Later, we established a categorization system of job-seeking behavior (SISC-CBE), where each job search behavior was assigned. With this categorization system, we created two variables. One variable referred to the presence/absence of each category in each of the five job search behaviors. The second collected the strategy the person used in each behavior. With these two variables, we measured the presence of the job search strategy. Basic descriptive statistical analyses were carried out to analyze the behavior, and the chi-square, contingency coefficient, Phi, and Cramer’s V, Lambda, and coefficient of uncertainty were calculated to study the differences.

All the statistical analyses were calculated using SPSS 22 software. Missing data on qualitative items represented about 0.1% of the sample. These participants were excluded from the analyses.

#### Results: Motivational and Behavioral Aspects of an Active Job Search by Gender

First, **Table 1** reports means and standard deviations by gender for motivational aspects of the active job search. For all groups, motivational aspects were medium-low. The lowest scores for men and women were for outcome expectations and role conflict, and the highest score was for internal locus of control.

**Table 1:** Descriptive statistics by gender for motivational aspects of an active job search.

	Men		Women	
		
	Mean	Standard deviation	Mean	Standard deviation
Unemployment attitude	3.35	2.01	3.65	2.02
Outcome expectations	1.99	0.70	2.68	7.82
Job search self-efficacy	3.12	0.51	3.10	0.46
Achievement motivation	3.24	0.50	3.21	0.53
Role conflict	2.28	0.52	2.28	0.28
Internal locus of control	3.59	0.86	3.55	0.92
External locus of control	2.94	0.63	3.04	0.64
Job control employment success	3.32	0.84	3.23	0.90


The F indicates that there were no significant differences between men-women. Results show that sex is not significant in the motivational aspects analyzed in the active job search (*F*_(2,236)_ = 290; *p* > 0.05). The tests carried out to analyze hypothesis 1 showed that women have the same motivation as men in the active job search. The results of the univariate ANOVA were not significant (unemployment attitude, *F*_(2,236)_ = 1.72, *p* > 0.05; outcome expectation, *F*_(2,236)_ = 0.46, *p* > 0.05; job search self-efficacy = *F*_(2,236)_ = 0.01, *p* > 0.05; achievement motivation *F*_(2,236)_ = 0.02. *p* > 0.05; role control *F*_(2,236)_ = 0.02, *p* > 0.05; internal locus of control *F*_(2,236)_ = 0.09, *p* > 0.05; external locus of control *F*_(2,236)_ = 0.05, *p* > 0.05; job control employment success *F*_(2,236)_ = 0.09, *p* > 0.05). They indicated that there were no significant gender differences in any of the motivational dimensions. Thus, Hypothesis 1 is confirmed.

Second, to analyze the job search behavior, the respondents were invited to write open-ended answers to questions about their job search behaviors. We collected a total of 389 terms. A content analysis was conducted. For categorization, the types or structures proposed in the past 10 years of research on job search were reviewed. These proposals were not useful, either because their design was confined to specific and restrictive coding approaches or because they were not appropriate for the functional approach used in this study. Although it is not possible to include here all the categorization criteria in the SISC-CVC for reasons of space, **Table 2** presents the general definition of each of the categories included in this coding system.

**Table 2:** Definition of categories: Categorization system for behavioral job search (CS-BJS).

General Category	Description
General search behaviors	Vague behaviors and without any details (e.g., “integrating me”).
Personal resources	Behaviors that develop objective and subjective aspects of the worker (e.g., “receiving training in any profession”) The sub-categories are: training, language learning, personal qualities development, job preferences, personal care, pre-work as voluntary (without pay), getting job experience in some profession.
Geographical mobility and labor flexibility	Behaviors of agreement about changing the workplace, the temporality of work activity, or the activities performed (e.g., “working at any time”).
Types of employment	Type of job and its regulation in the labor market (e.g., “being autonomous”). The sub-categories are: self-employed and official employee.
Search paths/information sources	Means of access to the job (e.g., “consulting employment exchange”). The sub-categories are: Networking (to develop and use contacts), employment agencies, employment services, temporary jobs, Social media and networking, company, employer websites, and notice boards.
Search tools	Behaviors that focus on presenting the tools used for the presentation of professional skills (e.g., “writing a curriculum correctly”). The sub-categories are: cover letter, curriculum vitae, interview, advertise myself and others
Search media	Ways to communicate with businesses (e.g., “searching on websites for businesses to call”). The sub-categories are: Internet, phone, and in person.
Timing/search frequency	Frequency of job search (e.g., “daily”). The sub-categories are: often, daily, weekly, and monthly.
Search strategies	Behaviors that focus on procedures to display skills (e.g., “working without being paid to show them how I work”).


The aforementioned specific indicators are shown in **Table 3** for each requested behavior in relation to its presence in job search behavior. The percentages by category and subcategory are also presented, according to the chosen strategy.

**Table 3:** Percentages of each category of job search methods.

	Behavior strategy 1		Behavior strategy 2		Behavior strategy 3		Behavior strategy 4		Behavior strategy 5	
					
	Men (%)	Women (%)	Men (%)	Women (%)	Men (%)	Women (%)	Men (%)	Women (%)	Men (%)	Women (%)
Personal resources	11.5	22.2	13.2	22.5	19.4	14.5	15.9	17.8	15	27.5
Training	4.4	11.5	5.3	7.8	8.7	5.6	6.3	8.3	7.5	15.9
Languages	0.9	1		3.9	2	1.1	-	1.2	-	2.9
Personal qualities	4.4	8.7	6.1	6.8	6.7	6.7	7.4	7.1	-	8.7
Job preferences	-	1	-	1	-	-	-	-	-	-
Personal care	0.9	-	0.9	1	1	-	1.1	-	7.5	-
Pre-work socializing habits	-	-	-	1	-	-	-	-	-	
Get job experience	0.9	-	0.9	1	1	1.1	1.1	1.2	-	
Geographical mobility and labor flexibility	3.5	0	0.9	0	4.8	4.4	5.3	4.8	2.6	2.9
Geographical mobility	3.5	-	0.9		3.8	3.3	5.3	4.8	1.3	2.9
Labor flexibility	-	-	-	-	1	1.1	-	-	1.3	-
Types of employment	0.9	1	1.8	1	0	3.3	2.1	1.2	5	2.9
Self-employed	0.9	-	1.8		-	1.1	2.1		5	-
Public employee	-	1	-	1	-	2.2	-	1.2	-	2.9
Search sources	27.3	18.2	41.2	9.8	25.3	39.7	48.6	41.7	54	37.6
Networking	11.4	4.8	17.5	13.6	2.2	7.8	31.6	26.2	32.5	11.6
Employment services	7	3.8	8.8	4.9	9.6	7.6	9.5	8.3	5	11.6
Employment agency	3.5	4.8	2.6	1	1.9	1.1	1.1	1.2	3.8	
ETT	-	1.9	2.6	-	-	4.4	1.1	-	-	1.4
Mass media	0.9	1.9	2.6	-	4.8	7.8	5.3	3.6	2.5	5.8
Social media and networking	1.8	-	0.9	-	1	2.2	-	-	3.8	-
Companies	0.9	-	4.4	2.9	4.8	5.5	-	1.2	3.8	4.3
Employer websites	1.8	1	0.9	1	1	3.3	-	1.2	1.3	2.9
Notice boards	-	-	0.9	-	-	-	-	-	1.3	-
Search tools	17.5	24.1	15.9	26.2	12.5	16.6	1.1	10.7	-	5.7
Cover letter	-	-	0.9			1.1	-		-	1.4
Curriculum	17.5	23.1	12.3	24.3	9.6	11.1	-	7.1	-	4.3
Interview	-	1	0.9	1.9	1	3.3	-	2.4	-	-
Advertise myself	-	-		-	1.9	1.1	1.1	1.2	-	-
Others	-	-	1.8	-		-	-	-	-	-
Media	36	32.7	16.6	12.7	13.5	12.2	11.6	13.1	8.8	8.6
Internet	23.7	12.5	9.6	11.7	8.7	6.7	8.4	9.5	2.5	7.2
Phone	-	-		1		1.1	2.1	2.4	2.5	
In person	12.3	20.2	7	-	4.8	4.4	1.1	1.2	3.8	1.4
General search behavior	3.5	2	9.6	1.9	5.7	11.1	6.4	10.7	2.6	10
Actively seek jobs	2.6	1	6.1	-	3.8	8.9	4.2	7.1	-	4.3
Search information	-	1	2.6	-	1.9	-	1.1	-	-	1.4
Nothing	-	-	0.9	-	-	-	-	-	1.3	4.3
Good means	0.9	-	-	-	-	-	-	-	-	-
Proper selection	-	-	-	1.9	-	-	1.1	3.6	-	-
No selection	-	-	-	-	-	2.2	-	-	-	-
Luck	-	-	-	-	-	-	-	-	1.3	-
Timing	0	0	0.9	1	1	0	0	0	0	1.4
Often	-	-	0.9	-	-	-	-	-	-	-
Daily	-	-	-	1	-	-	-	-	-	-
Weekly	-	-	-	-	1	-	-	-	-	-
Monthly	-	-	-	-	-	-	-	-	-	1.4
Search strategies	0	0	0	0	0	0	0	0	0	1.4
Show my skills	-	-	-	-	-	-	-	-	-	1.4


The data on the percentage of use of each strategy in job search behavior indicate that men mainly use search media and search sources, although they tend to use search tools as well. Women tend to use search sources and personal resources, whereas they use geographical mobility and labor flexibility very little (see **Table 4**).

**Table 4:** Percentages and degree of relevance of each category of explicit indicators of job search methods.

	Frequency (%)	
	
	Men	Women
Personal resources	44	58.2
Geographical mobility and labor flexibility	2.5	0
Types of employment	11	9.9
Search sources	77.8	69.4
Search tools	37.6	46.4
Media	68.1	64.2
General search behavior	63.2	55.5
Timing	1.7	1.8
Search strategies	0	0.9


Regarding the analysis of the different frequencies of use according to sex, the proportion of personal resources was higher for women (χ2 = 4.56; *p* < 0.05). Women use more behaviors to improve personal resources. At the same time, there are gender differences in the category of geographical mobility and labor flexibility (Likelihood ratio = 4.01; *p* < 0.05). Men use more behaviors related to geographical mobility and labor flexibility than women do. Thus, Hypothesis 2 is confirmed.

### Study 2: The Heterogeneity in the Female Group’s Experiences with Regard to Their Motivational and Job-Seeking Behavior Patterns

#### Participants and Procedure

The sample consisted of 235 women from the region of Valencia, Spain. The average age was 33.15 (*SD* = 11.66), and 45.1% had children. Participants had different educational levels [e.g., elementary school (29.8%) and university graduates (40.4%)]. Moreover, 42.1% received social benefits, and the respondents were primarily Spanish (71.9%). The sample was a convenience sample collected over a period of approximately 3 months in 2014. The selection criteria for the sample were as follows: Unemployed or precariously employed women who engaged in job seeking.

For this study, the same semi-structured interview as in study 1 was used to analyze the motivational and behavioral factors of the active job search.

#### Data analysis

Basic descriptive statistical analyses were carried out to obtain the means and standard deviations for the motivational variables. To analyze the job search behaviors, we used a free association test, as in Study 1. For the content analysis, we used the same categorization as in Study 1 (see **Table 2**). Two independent judges coded the items in selected categories. Consistency and reliability of the classification of job search strategies were obtained from the agreement between the two judges (Krippendorff, 1990, 2013). The Cohen’s Kappa value exceeded 0.82, indicating a high level of agreement (Altman, 1991). To analyze the behaviors, we created three variables, two categorical and one ordinal. The first categorical variable reflects the presence and/or absence of the strategy used in the five requested employment behaviors. The second categorical variable shows the strategy each woman used in each of the five behaviors (personal resources, geographical mobility, and labor flexibility, types of employment, search paths, tools, search media, search strategies, general, and timing). The third variable measures the frequency of use of each category of employment search behavior (**Table 2**) by each woman.

For the analysis of the women’s heterogeneity, we conducted a MANCOVA with the variables that Yerkes (2010) considered fundamental for the diversity of work. Due to the characteristics of the Spanish culture and the diversity of current family systems, the variable marital status and the presence/absence of children were substituted by the variable family responsibilities. This family responsibilities variable combines having children and having or not having a dependent family member, regardless of the woman’s marital status. We controlled the effect of age, nationality, and whether they obtained social benefits. Effect sizes were measured by Eta Squared (η2) values. η2 > 0.14 was not found to be relevant (Cohen, 1988).

All the statistical analyses were calculated using SPSS 22 software. Missing data on qualitative items represented about 0.1% of the sample. These participants were excluded from the analyses.

#### Results: The Heterogeneity of Experiences of the Female Group

As hypotheses 3 and 4 proposed, the results of the MANCOVA showed a multivariate effect for the motivational and behavioral aspects of an active job search in relation to family responsibilities (*p* < 0.001; η2 = 0.23) and university studies (*p* < 0.001; η2 = 0.25). The interaction between family responsibilities and university studies also influenced scores at the multivariate level (*p* < 0.001; η2 = 0.21).

Univariate analyses of the effect of family responsibilities on the motivational and behavioral aspects of the active job search showed significant predicted responses related to an internal locus of control (*F*_(6,235)_ = 5.82, *p* < 0.05) and job control employment success (*F*_(6,235)_ = 4.25, *p* < 0.05), with significantly more positive responses for women with no family responsibilities (internal locus of control *M* = 3.89; job control employment success *M* = 3.5) than for women with family responsibilities (internal locus of control *M* = 3.29; job control employment success *M* = 3.15).

Univariate testing for university studies of women found the effect to be significant for unemployment attitude (*F*_(6,235)_ = 9.44, *p* < 0.01, *M* = 3.32) and for frequency of use of personal resources (*F*_(6,235)_ = 11.36, *p* < 0.01, *M* = 2.47) and types of employment (*F*_(6,235)_ = 8.46, *p* < 0.01, *M* = 1.77). In this way, women with university studies scored higher in contrast to the non-college graduates (unemployment attitude *M* = 1.79; personal resources *M* = 1.76; types of employment *M* = 1.03). However, the women without university studies scored higher on job search self-efficacy (*F*_(6,235)_ = 5.71, *p* < 0.05, *M* = 3.32; university women *M* = 3.09) and frequency of use of information sources (*F*_(6,235)_ = 8.16, *p* < 0.01, *M* = 2.61; university women *M* = 2.05). Examination of the estimated mean indicated that the unemployment attitude was higher in university women than in non-university women, and that they used more personal resources, types of employment, and search strategies. However, they used fewer information sources and had less job search self-efficacy than the non-university women.

The interaction between university education and family responsibilities was significant. To examine the interaction, the score for unemployment attitude (*F*_(6,235)_ = 6.91, *p* < 0.01) was significantly greater in the university women with no family responsibilities than the scores for all the other women (non-college women with no family responsibilities *M* = 1.51; non-college women with family responsibilities *M* = 1.94; college women with no family responsibilities *M* = 3.53; college women with family responsibilities *M* = 2.1). Thus, Hypothesis 3 is no confirmed and Hypothesis 4 is confirmed.

## Discussion

The goal of this study was to analyze gender differences in motivations and behaviors of job search as a basic competency of employability in the Spanish context; in addition, we studied the heterogeneity and diversity of unemployed women motivation and job-seeking behavior. The results obtained showed that, as expected, there were no significant differences in motivational attributes by gender (hypothesis 1); the differences between men and women were observed in job search behaviors (hypothesis 2). Furthermore, there were some differences in women’s motivational (hypothesis 3) and behavioral (hypothesis 4) aspects depending on educational level and family responsibilities. These results have a number of theoretical and practical implications that we discuss in the following sections.

### Job Search by Gender

The advantage of the current study is that it proposes a model to integrate previous elements that have been studied separately and are relevant in explaining gender differences in the job search (motivational and behavioral aspects). This model has been tested in two samples: one sample composed of men and women and another one composed of only women. This has allowed us to corroborate the differences found and increase the knowledge about these differences. Future research could replicate the current findings in other vulnerability groups that, like women, can suffer the consequences of not considering their social context in their employability efforts.

Regarding motivational and behavioral job search by gender, some studies show no gender differences in the antecedents of job seeking (Van Hooft et al., 2005), and job search behavior varies between men and women (Kunze and Troske, 2015), as proposed by neoliberalism as well (Córdoba et al., 2013). Thus, the active job search by gender is not explained by motivational variables. Therefore, according to our results, personal resources are the strategy most preferred by women, in addition to some behaviors to balance the job search and family life (geographical mobility and labor flexibility). Our results showed that women used behaviors related to values associated with female differences and/or family cultures associated with their roles. In the current Spanish context, these asynchronies between motivation and behaviors are the traditional adscription of the household gender model (Beck-Gernsheim, 2003).

Our study can help to uncover the job search process in women and the mechanisms through which women have different behaviors from men. Our theoretical rationale and the results obtained point out that differences in behaviors are due to an attempt to balance work–family, as indicated by Hakim (2005). The consequences of maternity in women’s job search seem to be determined by the scarce redistribution of family responsibilities and the attribution to women of all the household responsibilities. This situation contributes to maintaining the women’s role (Bosch et al., 1999) and to lengthening their domestic role (Gartzia and Fetterolf, 2016). The assumptions of neoliberal politics have generated a model of the family where the State assumes that households must guarantee the members’ wellness. However, this is organized through the traditional division of roles (De la Cuesta and Bajo, 2006), where women have been incorporated into the labor market without a reduction in family tasks (childcare and care of other family members and housework).

Finally, in our study, the gender differences in job search behavior have been studied using a qualitative methodology. Our results approach the dimension of active job search proposed by Blau (1993); however, this dimension was evaluated with different multi-item behavioral measures. The contribution of the current study, using a free association test, is to broaden our knowledge of the job search gender gap. We hope our study contributes to enhancing the understanding of the perception and meaning of job search behavior. Future studies should also use this methodology to analyze the preparatory dimension proposed by Blau. Moreover, in relation to the job search behavior, the behaviors expressed in the free association test by women, and not by men, are related to controlled motivation (Deci and Ryan, 1985). In this context, it might be interesting to explore the linkage between autonomous and controlled motivation and job search behavior. In addition, the study of job search behavior with a free association test could be improved by studying the intensity and the effort people make. The information about behavior intensity has a positive relationship with the autonomous job search (Vansteenkiste et al., 2004), and people with high autonomous motivation could have better management of their job search behavior. Although intensity and effort have been related to employment (Van Hoye, 2013), effort assesses the amount of energy and time employed on job search behaviors, with no reference to a specific behavior. Therefore, studying the job search effort with a free association test could also improve the study of job search behavior.

In addition, our study is an initial empirical approach to a bioecological model of employability because it highlights the importance of the ecological context in the job search. We thought that research on a conceptual model of employability would help the research community to identify a useful framework and guide future research on employability (González-Romá et al., 2016). Considering this model, we have taken another step forward by applying it to studying the job search gap. The current labor system seems to be offering new job opportunities and economic autonomy to many women, but it assumes that the keys to success are personal resources, and people have internalized this idea. Nevertheless, this system deteriorates the access to and maintenance of employment, especially for women. The legitimizing discourse is based on women’s rights with an external view, but transversely it redefines these concepts in an individualistic manner related to the market, rather than as social and justice issues with the joint responsibility of societies, governments, and international institutions (Zabala, 2004). Moreover, the impact of neoliberal logic is highlighted, and it not only affects the economic sphere, but it also has repercussions in the social and culture spheres. The increasing fragmentation and individualization of collective actions produces a culture of the person that individualizes successes and failures related to the job world and does not pay attention to gender differences (Lechner, 1996). Therefore, our study adds new knowledge to the literature on gender in the job search, and future studies should analyze the interplay between individual-society to improve the understanding of the job search gap.

### Women’s Heterogeneity and Job Search

Second, regarding the heterogeneity of women, the present study found that there are motivational differences in women’s job search. Our findings show differences across educational levels and family responsibilities, as in Yerkes (2010). Then, university women without family responsibilities have higher unemployment attitudes when there is an interaction between university education and family responsibilities. We have used Hakim’s theory and role theory to explain this. In both frameworks, women’s heterogeneity is multi-causal, and we selected educational level and family responsibilities due to their connection with employability. Currently, a higher educational level in women has increased their knowledge and skills, producing changes in the job search (Choi et al., 2003). Carless and Arnup (2011) assert that the higher the women’s educational level, the more job search competences women have, and this affects motivational attributes. However, in the Spanish family culture (Flaquer, 2004; Moreno, 2010), women’s jobs are considered secondary, and they prefer jobs where they can balance work–family life (Gómez, 2008). This work-family role explains that there are no differences in job search behaviors among women.

The advantage of the current study is that it brings to light the gap in traditional gender roles. There are no individual differences or careers that discriminate between employed-unemployed woman (Formichella and London, 2005; García and Ibáñez, 2006). Women are solely responsible for their careers (Van der Heijde and Van der Heijden, 2006). However, some of them must increase their skills (Clarke, 2008), adaptability (McArdle et al., 2007), and proactivity (Fugate et al., 2004) in order to be employable. Thus, they must develop all those facets that make them more employable (Fugate et al., 2004), but the social context must address and reduce the gender gap.

In response to this situation, the ILO (2016) calls for efforts to manage the gender gap in access to employment. In this regard, our study has practical implications. Our findings suggest that women’s job search indicators are useful to analyze, explain, and propose psycho-social interventions. Two of the most important indicators are the educational level and work-family role. Liu et al. (2014), in a meta-analytic study, examined the effectiveness of job search interventions, and they found that programs are effective when they include improvements in job search skills, self-presentation, self-efficacy, proactivity, goal setting, and social support. Nevertheless, our results do not find behavior differences among women. In order to achieve employment success, intervention programs have to focus on training and changing behavior related to women’s social roles.

Furthermore, our results show that the gender role is present in social representations and warrants reflection by all stakeholders. It is important to build a harmonious, equitable, and inclusive job context. This process would have significant gender effects because it would develop a new cultural view of the balance between work–family life (Farah and Salazar, 2009).

In addition, our study has other implications; in order to find out whether programs and stakeholders’ reflections are effective, it is necessary to have information about job search outcomes. Thus, future studies should provide empirical evidence about the two-dimensional model of job search proposed by Saks et al. (2015) based on gender, in other words, job search behavior and job search outcomes. Job search outcomes have received little attention, and so more studies are needed (Lim et al., 2016).

Finally, the research on gender brings to light a work-family conflict, as Hoffman and Moon (2000) state. It shows that there are still invisible discrimination mechanisms that hinder women’s careers and leave them in a situation of inequality at work. Therefore, in this study we analyze this contradiction and point out that political issues lead women to conditions of inferiority and vulnerability (Farah and Salazar, 2009). Therefore, our results have important theoretical and practical implications at the political level. The gender gap needs public support (Johnston and Diekman, 2015); namely, the State must regulate and act to eliminate the gender gap, helping to develop effective intervention programs to employ women and promote gender equity.

## Limitations

Our study has a number of limitations that must be kept in mind. First, all the data were collected from the same source in the form of individual perceptions. This fact might have inflated the observed relationships among the variables due to common-source bias. However, we used two samples to study the motivational and behavioral job search. First, we studied the gender gap in these variables, and then we studied them using a sample of women. This approach can increase our knowledge about the job search in women, even though the same source of information was used. Moreover, we used two kinds of methodologies to collect the information. We used both questionnaires and a free association test. They provided more knowledge about the same issue. Second, the Cronbach alpha values for one of the factors (internal attribution) were below the acceptable level of internal consistency. However, as Lester et al. (2014) state, the magnitude of the coefficient is directly related to the number of items on a test, and a shorter test may be acceptable with coefficients of 0.60 or in the low 0.70s. In addition, Loewenthal (1996) suggests that a reliability value of 0.6 or close to it can be considered acceptable for scales with less than 10 items. Furthermore, as a general criterion, George and Mallery (2003) suggest that a coefficient alpha close to 60 is questionable, but not unacceptable. Guilford (1965) explains that a reliability of 0.50 is sufficient for basic research. Even some authors such as Schmitt (1996) report that measures with relatively low reliability can be very useful because very high coefficients may indicate excessive redundancy in the items (very repetitive). Third, the cross-sectional design does not allow us to evaluate the developmental trajectories or the causal links. However, this study, as we mentioned above, is an initial empirical approach to a bioecological model of employability. We can highlight the importance of the ecological context in the job search, and although a longitudinal methodology would analyze possible changes in job-seeking based on gender-related family life events (e.g., marriage, having children, and responsibility for older parents), we first need to collect the behaviors using a qualitative methodology like the one used in this study.

## Conclusion

The current research highlights the importance of job search gender differences. Findings indicated that there are gender differences in job search behaviors, but in women, job search differences are mainly based on motivational aspects. Understanding this situation increases knowledge about the gender gap, allowing us to become aware of the social changes needed to improve employability. We hope our study contributes to pointing out indicators of effectiveness in training programs for women’s employment. Furthermore, we intend to promote critical reflection about how the social and labor context justifies structural gender inequalities by using an individualist and functionalist discourse.

## Ethics Statement

Ethical approval was granted by the Human Research Ethical Committee of the Experimental Research Ethics Commission, University of Valencia.

## Author Contributions

LL-I and PG-N reviewed the literature, made the introduction, analysis and results and wrote the discussion, taking into account the different references used in the paper. AC-I extracted the information to revise the discussion and the final English version. JZ-G reviewed the literature and selected the information and he revised the discussion and the final English version.

## Conflict of Interest Statement

The authors declare that the research was conducted in the absence of any commercial or financial relationships that could be construed as a potential conflict of interest.
